# Dr. E. Brown-Séquard

**Published:** 1860-07

**Authors:** 


DR. E. BROWN-SÉQUARD has been appointed chief physician to the "National Hospital for the Paralyzed and Epileptic," a new charity recently established in London. He has already commenced a course of clinical lectures on the diagnosis and treatment of the principal forms of paralysis of the lower extremities, and will prove an ornament to the profession of the British metropolis. The publication of the *Journal de Physiologie* will not be affected by his removal, but will appear as heretofore in Paris, having been placed under the direction of M. Balbiani.

